# Patient Experience in Health Center Medical Homes

**DOI:** 10.1007/s10900-015-0042-0

**Published:** 2015-05-31

**Authors:** Nicole Cook, Lucas Hollar, Emmanuel Isaac, Ludmilla Paul, Anthony Amofah, Leiyu Shi

**Affiliations:** Master of Public Health Program, College of Osteopathic Medicine, Nova Southeastern University, 3200 South University Drive, Fort Lauderdale, FL 33328 USA; Broward Community and Family Health Centers, 5010 Hollywood Boulevard, Suite 100-B, Hollywood, FL 33201 USA; Health Choice Network of Florida, 9064 NW 13th Terrace, Miami, FL 33172 USA; Health Policy and Health Services Research, Johns Hopkins Bloomberg School of Public Health, Hampton House, 624 N. Broadway, Baltimore, MD 21205 USA

**Keywords:** Patient experience, Federally qualified health center, Patient centered medical home, Quality improvement, Underserved populations

## Abstract

The Human Resource and Services Administration, Bureau of Primary Health Care Health Center program was developed to provide comprehensive, community-based quality primary care services, with an emphasis on meeting the needs of medically underserved populations. Health Centers have been leaders in adopting innovative approaches to improve quality care delivery, including the patient centered medical home (PCMH) model. Engaging patients through patient experience assessment is an important component of PCMH evaluation and a vital activity that can help drive patient-centered quality improvement initiatives. A total of 488 patients from five Health Center PCMHs in south Florida were surveyed in order to improve understanding of patient experience in Health Center PCMHs and to identify quality improvement opportunities. Overall patients reported very positive experience with patient-centeredness including being treated with courtesy and respect (85 % responded “always”) and communication with their provider in a way that was easy to understand (87.7 % responded “always”). Opportunities for improvement included patient goal setting, referrals for patients with health conditions to workshops or educational programs, contact with the Health Center via phone and appointment availability. After adjusting for patient characteristics, results suggest that some patient experience components may be modified by educational attainment, years of care and race/ethnicity of patients. Findings are useful for informing quality improvement initiatives that, in conjunction with other patient engagement strategies, support Health Centers’ ongoing transformation as PCMHs.

## Background and Significance

The Human Resources and Services Administration (HRSA) funded Health Center program provides comprehensive, primary care services for more than 22 million patients, many of whom are un or underinsured and from medically underserved populations. Health Center program funding is contingent upon Health Centers having an ongoing quality improvement and quality assurance plan that supports high quality patient care, as well as a governing board which is comprised of a majority of consumers [1]. As such, Health Centers have traditionally embraced a culture of both quality improvement and patient engagement, two fundamental components, among other attributes, that align Health Centers with the Patient Centered Medical Home (PCMH) model [2].

With HRSA support, many Health Centers were early adopters of national initiatives to improve care, including electronic health records and transformation to achieve designation as PCMHs [3]. The PCMH model aligns with the Institue of Healthcare Improvement’s Triple Aim goals of improving the experience of care, improving the health of populations, and controlling health care costs which, when addressed together, are anticipated to improve health care in the United States [4]. For Health Centers who achieve PCMH designation or recognition, the meeting or exceeding of PCMH accreditation standards is only one important step along the transformative process. Health Centers that are best positioned to contribute to the Triple Aim must adopt a culture of “medical homeness” that continuously assesses and evaluates their care model in terms of experience, quality and cost.

While cost and quality have been at the forefront of PCMH evaluations, measurement of patient experience has received less attention [5]. In PCMHs, patient experience should be evaluated regularly to understand the impact of transformation activities and to inform on quality improvement initiatives [6, 7]. Patient experience refers to how patients perceive the care they receive. Through patient experience feedback, patients inform Health Centers about which areas of care access, delivery and coordination are concerns for them and their suggestions for improvement. This feedback, in conjunction with other patient engagement initiatives, supports quality improvement priority setting and initiatives that are specifically patient-centered, which, in turn, are anticipated to improve health care outcomes [8–10]. Though patient experience measures, by nature, are subject to bias [11], as they measure perception of care rather than actual care delivered or received, patient experience may also serve as robust measures of quality care delivery [9, 12, 13].

Research on patient experience of care in Health Centers is limited, but growing. A nationally representative study of Health Center patients in 2009 revealed that more than 80 % of patients rated the quality of care they received at Health Centers as “very good” or “excellent” [14–16]. While the study did not differentiate patients from Health Centers designated as PCMHs, survey results were stratified by patients who scored their Health Center high on an number of PCMH-related attributes, including those elements related to access to care, communication and self-management support for chronic conditions [14]. Results showed that patients who scored their Health Centers higher on PCMH-related attributes had higher odds of reporting better quality of care; suggesting that PCMH attributes may influence better experience. A 2012 study across 26 safety-net clinics in New Orleans suggested that patients’ experience of care coordination was better among those clinics that had implemented more PCMH improvements [17]. However, there was no difference in reported access to care or confidence in quality/safety of care by PCMH improvements.

As of 2015, nearly 60 % of all Health Centers have achieved PCMH recognition through demonstrating to national accrediting bodies that they are committed to providing primary care that is comprehensive, patient-centered, coordinated, and accessible, and focused on quality [18]. While there are no current studies assessing the relationship between Health Center PCMHs and patient experience, results from other studies conducted with primary care populations is mixed in terms of how PCMH may influence patient experience [19–25].

The present study contributes to our understanding of patient experience in Health Center PCMHs. In addition, this study also identifies opportunities for quality improvement that Health Centers can consider as they continue their journey of PCMH transformation.

## Methods

### Overview

We conducted a cross-sectional survey of adult patients of five Health Centers in Broward and Miami-Dade counties, Florida, each of which had received designation as a PCMH by the National Committee for Quality Assurance at least a year prior to surveying. The five Health Centers served a combined patient population of more than 162,000 in 2013, of whom 63 % were uninsured, 85 % were below 200 % of the federal poverty level, 39 % were black and 51 % were Hispanic. Thirteen Health Center sites in total were included in the study (three sites from each of four Health Centers and one site from one Health Center). All Health Centers in this study are members of Health Choice Network, Inc. (HCN) a health-centered controlled network that provided technical assistance to assist members in preparing for PCMH designation through a Center of Excellence for PCMHs.

### Data Collection and Study Participants

The institutional review board at Nova Southeastern University reviewed the study and deemed it exempt.

Patients were surveyed between February 2014 and April 2014. All surveys were conducted face-to-face at the Health Centers by faculty and students from a Master of Public Health program. As many surveyors were multi or bilingual, patients were surveyed in their chosen language of English, Spanish or Haitian Creole. Survey data was collected in Snap Survey Software (Snap Surveys, Portsmouth, New Hampshire) installed on iPads (Apple, Cupertino, California).

Eligible patients included adults 18 years or older who were at the Health Center for a visit for themselves and who also had a previous visit during the past 12 months. Patients who met the screening criteria were asked if they would complete a short survey about their experience with receiving care. Patients were advised that they would receive a $5 Wal-Mart gift card for completing the survey. More detail regarding data collection can be found elsewhere [24].

### Measures

Initially, the project team reviewed a number of patient satisfaction and patient experience tools including the Clinician and Group Surveys Consumer Assessment of Healthcare Providers and Systems (CG-CAHPS) and the CG-CAHPS PCMH item set, as well as other tools and questions that were available in peer-reviewed manuscripts or were made available by authors [16, 17, 20, 22]. As many of the Health Centers indicated they planned to use CG-CAHPS in the future, efforts were made to select questions from this tool so that Health Centers would be able to use collected data as a benchmark for future patient experience surveys.

The NSU project team developed an initial question set of 36 questions which was vetted to the three Health Center partners and three health-system ambulatory care partners who were concurrently participating in a patient experience evaluation. Partners were asked to select the 12–15 questions that were of highest priority for them. They were also asked to suggest revisions to proposed questions and to suggest new questions. Project team members worked together to collate results and develop a revised draft tool which was vetted again with partners. The entire process of revision and vetting was repeated a third time. The final vetted tool was then pilot tested with four patients, which resulted in some additional minor revisions to wording. The survey and screening questions were then translated and back translated in Spanish and Haitian Creole by native language speakers. The final survey included nine demographic questions, three questions addressing health status, 12 questions addressing patient experience, two questions regarding accessibility of medical information, one question to assess perceived change in care over the past 2 years and two questions intended specifically to inform on opportunities for improvement (“name one thing you like about getting care here” and “name one thing you would improve about getting care here”). The 12 patient experience questions covered three patient experience domains: patient centeredness, coordinated care and access to care. Except those open-ended questions intended to inform on specific opportunities for improvement, all patient experience questions included categorical responses (“yes”, “no,” “not sure”) or ordinal responses (“never”, “sometimes”, “usually”, “always”). Though all patient experience questions were close ended, patients were able to provide commentary. All commentary was recorded by the data collectors.

### Analysis

There were a total of 505 surveys collected from unique patients at the Health Centers. The targeted goal, based on resource allocation, was to collect a minimum of 100 surveys from each Health Center, distributed equally among the number of sites per Health Center surveyed. Of the 505 surveys collected, 17 surveys were not included in the final study file of 488 due to missing data or outlier data (e.g. age = 3) in one or more field included in the analysis.

Descriptive statistics were used to describe the surveyed population in terms of demographic characteristics and health status. Bivariate analyses were performed to compare patient experience measures across demographic characteristics. To assess predictors of better patient experience we conducted multivariate modeling using binary logistic regression to investigate associations between better patient experience and patient characteristics (demographic characteristics, smoking and having a health condition). Only those patient experience questions that had significant associations with the demographic variables in the bivariate analyses were included in the regression modeling (questions 1, 2, 5–11).

Patient characteristics, the independent variables, were collapsed into categories to support interpretation of bivariate and multivariate analyses as follows: age (0 = <45; 1 = ≥45); years of care (0 = <3; 1 = ≥3); language (0 = no spoken English; 1 = speaks English); Race (0 = Black; 1 = White; 3 = other, which includes two or more races and unreported); Ethnicity (0 = Hispanic, 1 = African American, 2 = Caribbean Islander, and 3 = other and unreported); health condition (0 = no reported health condition; 1 = any reported health condition). With regards to the patient experience questions, the dependent variables in the bivariate and multivariate analyses, the most positive selection was coded as 1 (e.g. “always” or “yes”) and all other responses were collapsed into 0.

Data was analyzed in SPSS (version 23, Chicago, Illinois).

## Results

Table 1 summarizes the population of 488 patients surveyed. The average age of respondents was 44.5. More than 65.0 % of patients had been patients at the Health Center for at least 2 years. Respondents were more likely to be women (75 %). More than 42 % of respondents were Black and 48.0 % were Hispanic. In terms of education, the majority of patients were High School graduates. Overall, 59.6 % or patients reported they had Hypertension, 19.9 % Diabetes, 22.3 % were overweight or obese and 12.9 % were smokers. The average stress rating by patients on a scale of 1–10 was 5.1.

**Table 1:** Characteristics of health center patients who completed patient experience surveys (n = 488)

	No.	%
Age (mean, SD)	488	44.5 (14.8)
Years of care		
1	171	35.0
2–5	198	40.6
6 or more	119	24.4
Gender		
Male	117	24.0
Female	370	75.8
Transgender	1	0.20
Marital status		
Single	206	42.2
Married	177	36.3
Divorced	63	12.9
Separated	26	5.3
Widowed	15	3.1
Not sure	1	0.2
Languages spoken^a^		
English	364	74.6
Spanish	250	51.2
Creole	97	19.9
French	48	9.8
Race		
Black	208	42.6
White	194	39.8
Two or more/other	60	12.3
Not sure	26	5.3
Ethnicity		
Hispanic	234	48.0
African American	117	24.0
Caribbean Islander	93	19.1
Other/not sure	44	9.3
Education		
No HS grad	102	20.9
HS grad	279	57.0
College grad	107	21.9
Reported health conditions		
Hypertension	196	59.6
Diabetes	97	19.9
Asthma	58	11.9
Heart disease	33	6.8
Overweight/obese	109	22.3
Depression	93	19.1
Current smoker	63	12.9
Stress scale 1–10 (mean, SD)^b^	480	5.1 (2.7)

^a^Patients could report multiple responses. As a result, total # in category may equal more than 488

^b^Eight subjects responded “not sure” as to stress level

Responses to the patient experience questions, by domain, are listed in Table 2. Overall, patients responded favorably (range of “always” responses 79.1–87.7 %) to patient centeredness questions including being treated with courtesy and respect (Q1), helpfulness of clerks, receptionists and other office staff (Q2), time spent with their provider (Q3), and provider communication (Q4). Responses varied across coordinated care domain questions with nearly 87 % of patients reporting that they receive reminders about tests, treatments or appointments from their Health Center (Q7) to 23.6 % of patients with at least one reported health condition indicating that they had been referred or recommended to a workshop or educational program to take better care of their health (Q9). Responses in the access to care domain were lower with only 53 % or patients indicating that it is easy to contact their provider via phone (Q10) and 51.4 % indicating they could get an appointment as soon as they needed one (Q11). Patients were asked if they prefer another method besides the phone to make appointments or get medical advice (Q12). Among the 36.7 % who indicated “yes”, 46.4 % said they prefer email, 21/2 % prefer text, 8.9 % prefer a letter and 6/1 % said they would like to come in person to the Health Center.

**Table 2:** Patient experience among health center patients in South Florida (n = 488)

	% “Always”	% “Yes”
*Patient centeredness*		
Q1. In the past year, how often did clerks, receptionists and other office staff at your provider’s office/Health Center treat you with courtesy and respect?	85.0	
Q2. In the past year, how often were clerks, receptionists and other office staff at your provider’s office/Health Center helpful to you in terms of scheduling appointments, answering questions, and getting referrals?	79.1	
Q3. In the past year, how often did your healthcare provider spend enough time with you?	79.3	
Q4. In the past year, how often did your provider respond to your health questions or concerns in a way that was easy to understand?	87.7	
*Coordinated care*		
Q5. In the past year, did anyone in your provider’s office/Health Center work with you to make/create specific goals for you?		60.2
Q6. In the past year, did you and anyone in your provider’s office/Health Center talk at each visit about [any and] all the prescription medicines you were taking?		78.2
Q7. In the past year, did you get any reminders about tests, treatments or appointments from your provider’s office/Health Center between visits?		86.9
Q8. In the past year, did your provider follow up with you about results of blood tests, X-ray, or other tests in between visits?		68.2
Q9. In the past year, did anyone in your provider’s office/Health Center refer/recommend a workshop or education program to help you take better care of your health?^a^		23.6
*Access to care*		
Q10. How easy is it to contact someone at your provider’s office/Health Center over the telephone about a health problem during regular office hours?^b^		53.0
Q11. In the past year, when you made an appointment for a check-up or routine care with your provider/Health Center, how often did you get an appointment as soon as you needed?		51.4
Q12. Do you prefer another method of contact besides phone to make appointments and/or get medical advice?^c^		36.7

^a^Among patients with reported health condition (smoker, obese/overweight, hypertension, diabetes, asthma, heart disease or depression), n = 313

^b^22 not applicable

^c^Email—83 (46.4 %); Text—38 (21.2 %); Letter—16 (8.9 %); In person—11 (6.1 %)

Results of the bivariate analysis are included in Table 3. Overall there were few significant relationships between patient characteristics and patient experience. Years of care was significantly associated with being treated with courtesy and respect (Q1) and talking about prescriptions at each visit (Q6). Race and ethnicity were significantly associated with several questions in the coordinated care domain and education was significantly associated with questions related to access to care. There were no significant relationships between demographic variables and the time the provider spends during the visit (Q3) or provider communication in a way that is easy to understand (Q4).

**Table 3:** Comparison of patient experience responses by characteristic of health center patients (n = 488)

	Q1. Treated with courtesy and respect	Q2. Office staff helpful	Q3. Provider spend enough time	Q4. Provider respond in way easy to understand	Q5. Create specific goals	Q6. Talk about prescriptions at each visit	Q7. Reminders about tests, treatments or appointments	Q8. Follow-up about results	Q9. Recommend workshop or education program^a^	Q10. Easy to contact over telephone	Q11. Get an appointment as soon as needed
Age	0.37	0.74	0.83	0.34	0.23	<.01**	0.04*	0.21	1.00	0.40	0.47
Years of care	0.02*	0.22	0.58	0.10	0.14	<.01**	0.02*	0.03*	0.79	0.08	0.32
Gender	0.14	0.24	0.15	0.26	0.91	0.61	0.88	0.07	0.88	0.59	0.34
Language	1.00	1.00	1.00	1.00	0.46	0.71	1.00	0.58	0.05	0.02*	0.10
Race	0.87	0.33	0.12	0.13	<.01**	0.11	0.03*	0.04*	0.45	0.84	0.24
Ethnicity	0.96	0.36	0.05	0.26	<.01**	0.26	0.22	<.01**	0.01*	0.19	0.26
Education	0.65	0.04*	0.12	0.64	0.61	0.93	0.92	0.87	0.63	<.01**	<.01**
Smoker	0.45	0.87	0.40	1.00	0.58	1.00	0.43	0.15	0.01	0.33	0.36
Health condition	0.89	0.56	0.10	1.00	0.12	<.01**	0.67	0.26	–	0.92	0.78

*P* value calculated to assess differences in question responses (column by patient characteristics row). Chi square tests for categorical variables

* *P* < 0.05

** *P* < 0.01

^a^Among subset of patients with a reported health condition (n = 313) as it may not be indicated to send patients without reported health condition to workshop of education program

When analyzed in the multivariate models, there were few differences in patient experience by most independent variables. Patients with more years of care were less likely to respond favorably to being treated with courtesy and respect (Q1) or ease of contacting someone over the telephone (Q10). However, they were more likely to respond that their provider “always” talks to them about prescriptions at each visit (Q6). Patients with college degrees were less likely to find office staff helpful (Q2). They were also less likely to respond that the office was easy to contact over the telephone (Q10) and that they could get an appointment as soon as they needed one (Q11). Among a subset of patient with a health condition, smokers were more likely to be referred to attend a workshop or educational program (Q9). Patients with a reported health condition were more likely to speak to their doctors about their prescription at each visit (Q6). In terms of race and ethnicity, white patients were significantly more likely to talk about their prescriptions at each visit (Q6) and to report that they received follow-up about results compared to black patients (Q8). Patients of other race (including two or more races, unknown and unreported) were less likely to report that someone in the Health Center had worked with them to create specific goals (Q5) compared to black patients. Compared to Hispanic patients, African American patients were significantly more likely to report that they received follow-up about results (Q8), and patients of Caribbean Islander ethnicity were more likely to report that they talked about prescriptions at each visit (Q6) (Table 4).

**Table 4:** Adjusted models predicting patient experience for select questions by characteristics of health care patients (n = 488)

	Q1. Treated with courtesy and respect	Q2. Office staff helpful	Q5. Create specific goals	Q6. Talk about prescriptions at each visit	Q7. Reminders about tests, treatments or appointments	Q8. Follow-up about results	Q9. Recommend workshop or education program^a^	Q10. Easy to contact over telephone	Q11.Get an appointment as soon as needed
Age									
< 45 (ref)									
=>45	0.90 (.50–1.62)	1.18 (0.71–1.98)	1.03 (0.67–1.58)	1.40 (0.83–2.35)	1.62 (0.86–3.05)	1.18 (0.74–1.87)	1.18 (.61–2.04)	0.80 (0.52–1.23)	1.07 (0.70–1.63)
Years of care									
< 3 (ref)									
=>3	0.55 (0.32–0.94)*	0.64 (0.40–1.03)	1.22 (0.83–1.81)	1.69 (1.06–2.71)*	1.59 (0.89–2.84)	1.35 (0.88–2.05)	1.03 (.58–1.84)	0.66 (0.44–0.98)*	0.75 (0.51–1.11)
Gender									
Male (ref)									
Female	1.60 (0.89–2.85)	1.33 (0.78–2.26)	1.02 (0.64–1.62)	1.03 (0.59–1.80)	1.19 (0.61–2.30)	0.70 (0.42–1.17)	0.96 (.51–1.79)	0.70 (0.44–1.11)	0.69 (0.44–1.08)
Language									
No English (ref)									
Speaks English	0.89 (0.45–1.74)	0.98 (0.55–1.75)	1.11 (0.68–1.82)	0.96 (0.53–1.72)	1.29 (0.62–2.65)	1.19 (0.71–2.00)	1.6 (.72–3.58)	0.65 (0.37–1.08)	0.65 (0.40–1.05)
Race									
Black (ref)									
White	0.98 (0.26–3.64)	1.34 (0.42–4.32)	0.79 (0.30–2.07)	3.63 (1.07–12.31)*	1.65 (0.40–6.69)	3.34 (1.18–9.42)*	3.22 (.70–14.82)	0.81 (0.29–2.25)	0.85 (0.33–2.22)
Other	1.28 (.047–3.49)	1.32 (0.40–4.37)	0.34 (0.13–0.91)*	2.16 (0.62–7.50)	0.61 (0.15–2.41)	2.01 (0.70–5.78)	1.86 (.41–8.54)	0.69 (0.24–2.00)	0.84 (0.32–2.24)
Ethnicity									
Hispanic (ref)									
African American	1.15 (0.29–4.60)	1.89 (0.55–6.57)	1.12 (0.40–3.14)	3.09 (0.86–11.07)	0.75 (0.17–3.30)	3.42 (1.11–10.53)*	4.63 (.95–22.58)	0.57 (0.20–1.68)	1.07 (0.39–2.96)
Caribbean Islander	1.14 (0.30–4.35)	1.69 (0.50–5.67)	1.10 (0.41–2.98)	4.96 (1.36–18.12)*	0.44 (0.11–1.82)	0.80 (0.28–2.30)	1.67 (.36,7.65)	0.66 (0.23–1.92)	1.26 (0.47–3.37)
Other	1.28 (.047–3.49)	0.95 (0.42–2.14)	0.66 (0.33–1.33)	0.80 (0.36–1.80)	0.68 (0.24–1.93)	0.63 (0.30–1.34)	0.68 (2.44–1.91)	0.50 (0.24–1.03)	0.79 (0.39–1.60)
Education									
No HS degree (ref)									
HS degree	0.97 (0.48–1.94)	0.71 (0.37–1.35)	0.88 (0.53–1.46)	1.06 (0.58–1.92)	0.90 (0.43–1.85)	0.92 (0.54–1.58)	0.92 (.46–1.85)	0.85 (0.52–1.42)	0.68 (0.42–1.12)
College degree	0.73 (0.32–1.64)	0.44 (0.21–0.92)*	0.85 (0.46–1.56)	1.23 (0.60–2.54)	0.89 (0.37–2.16)	0.87 (0.46–1.67)	0.81 (.33–1.99)	0.40 (0.22–0.75)**	0.35 (0.19–0.64)**
Smoking status									
Nonsmoker (ref)									
Current smoker	1.36 (0.56–3.23)	1.01 (0.49–2.10)	1.21 (0.66–2.24)	0.85 (0.41–1.75)	0.59 (0.26–1.38)	1.17 (0.59–2.31)	2.02 (1.05–3.87)*	1.32 (0.71–2.46)	1.29 (0.71–2.32)
Health condition									
No (ref)									
Yes	1.12 (.061–2.07)	1.06 (0.62–1.81)	1.19 (0.75–1.87)	1.76 (1.04–3.00)*	1.10 (0.57–2.12)	1.10 (0.68–1.79)		1.02 (0.64–1.62)	0.90 (0.58–1.42)

Logistic regression; OR and 95 % confidence interval. ORs are considered significant if the 95 % CI does not include 1.0

* *P* < 0.05

** *P* < 0.01

^a^Model includes subset of patients with a reported health condition (n = 313) as it may not be indicated to send patients without reported health condition to workshop of education program

## Discussion

To our knowledge, this is the first study to report patient experience among Health Centers recognized as PCMHs by accrediting bodies. The positive experience reported by Health Center patients, specifically in terms of patient centeredness, is not surprising as both the mission and program requirements for Health Centers emphasize quality improvement and patient engagement. Prior reported experience among Health Center patients has been demonstrated to be positive, particularly in terms of accessibility and communication, with few reported disparities in terms of race, ethnicity or insurance [14, 16]. Though Health Centers care for populations that historically face greater disparities with regards to access to care, research has shown that Health Centers provide cost-effective quality care that is often comparable to non-Health Center primary care providers [26–29].

While the population of patients in this study included a convenience sample of patients of five Health Centers in south Florida, the population is diverse in terms of race, ethnicity and educational attainment. As we only surveyed adult patients, the high percent of patients with health conditions, including hypertension, was not surprising.

Though patients reported very positive experience with patient centeredness and some coordinated care components including talking about prescriptions (Q6) and receiving reminders (Q7); results suggest several opportunities for priority setting that may translate into quality improvement initiatives, including improving goal setting (Q5), referral/recommendation to a workshop or education program (Q9), contacting someone at the Health Center via phone (Q10), improving appointment availability (Q11) and increasing communication methods (such as by using email and text). Several of these findings, particularly with regards to referral to workshops and educational programs and improving appointment available have been previously reported by disadvantaged primary-care patients as priorities that need to be addressed by health care systems [30]. Results of bivariate and multivariate analyses emphasize that quality improvements initiatives would best be developed with involvement of a diverse group of patients in terms of years of care as a Health Center patient, race, ethnicity, and educational attainment, as experience did vary significantly by some characteristics.

As it is widely recognized that PCMH is a transformative process and that ongoing assessment of patient experience can help to inform on specific areas that should be integrated into ongoing quality improvement initiatives [8, 10]. In this study, opportunities identified through patient experience assessment are being addressed by initiatives at both the Health Center and network level. Strategies being evaluated and implemented across the Health Centers include pre-planned visits where medical home coordinators contact patients prior to their visit to review labs and tests, morning huddles where the care teams review specific needs for patients who are scheduled for that workday, extended evening hours, centralized call centers, providing assistance to patients to register for the electronic health record patient portal, providing “walk-in” slots for patients needing same day appointments, and other quality improvement initiatives. From an HCN network perspective, initiatives implemented at the Health Centers included in this study and among the other 26 Health Centers members are shared and discussed regularly at monthly Clinical and Quality Improvement Committee meetings and quarterly through a Health Care Quality Institute, a national forum to share promising and best practices. Patient engagement activities including ongoing satisfaction and experience surveys, focus groups, and participating in research committees to drive appropriate health services research.

This study had several limitations. First, results may be limited by response bias, which can be introduced when using a convenience sample. Though the sample is diverse, it is possible that an important group of Health Center patients may not have been included. Furthermore, results represent patients from Health Centers in Broward and Miami-Dade County and, as such, findings may not be generalizable to Health Centers outside the area. Second, survey research may be subject to recall bias, social-response bias and interview bias. Efforts were made to reduce these biases through surveyor training, development of scripts and interview protocols and notifying patients that surveyors represented NSU, not the Health Center. However, the extent to which bias was introduced was not independently assessed. Third, some variables which may be important to understanding access and coordination of care, including health insurance, income, employment and transportation, were not included in the survey. Future studies on patient experience should consider a greater representative sample of Health Center medical homes, incorporation of Health Center-level organizational and operational variables that may confound our understanding of patient experience (for example, organizational culture, staffing models and provider productivity) as well as patient-level variables that may be associated with experience. Given that most Health Centers have sought, or are currently in the process of seeking, PCMH designation, conducting more robust study designs, such as controlled trials, may be somewhat limited. Nonetheless, in order to continue to evaluate the role of PCMH transformation on patient experience, future study designs should be longitudinal in nature.

This study adds to our understanding of patient experience in Health Center PCMHs. Overall, patients reported that their Health Centers are patient-centered, that they are treated with courtesy and respect and that their provider responds to their health questions in a way they can understand. Opportunities identified by patients include improving ability to contact the Health Center by phone, appointment scheduling, follow-up about tests and referral to health-related workshops or education programs. Health Centers included in this study are currently addressing these findings through ongoing quality improvement initiatives, which is a critical element of the patient experience assessment process as Health Centers continue along their journey of PCMH transformation. Though Health Centers have traditionally maintained a culture of patient engagement and quality improvement, it is now more important than ever to develop systems of quality care that meet patients’ needs. As previously uninsured Health Center patients are now insured through provisions in the Affordable Care Act, they will likely have a greater choice in where they can receive care. Through ongoing assessment and engagement of patient experience, Health Centers can continue to position themselves to emerge as “providers of choice” for patients and valuable players in driving the Triple Aim for our nation’s health.
